# Extra-uterine low grade endometrioid stromal sarcoma arising from ovarian endometriosis: a case report and review of the literature

**DOI:** 10.1186/s40661-019-0067-7

**Published:** 2019-01-29

**Authors:** Boubacar Efared, Ibrahim S. Sidibé, Fatimazahra Erregad, Nawal Hammas, Laila Chbani, Hinde El Fatemi

**Affiliations:** 1 Department of pathology, Hassan II University Hospital, Fès, Morocco; 2 Department of pathology, FSS, UAM, Niamey, Niger; 3 Laboratory of Biomedical and Translational Research, Faculty of Medicine and Pharmacology, Sidi Mohamed Ben Abdellah University, Fès, Morocco

**Keywords:** Endometrioid stromal sarcoma, Uterus, Ovary, Endometriosis

## Abstract

**Background:**

Endometrial stromal sarcoma (ESS) is a rare neoplasm accounting for only 0.2% of female genital tract tumors. The primary extra-uterine location of ESS is an extremely uncommon occurrence.

**Case presentation:**

We present a case of a 64-year-old woman presenting with abdominopelvic and bilateral ovarian tumors with misleading clinical presentation and diagnostic challenge. The histopathological examination of the resected specimens disclosed the diagnosis of primary extra-uterine ESS arising from ovarian endometriosis. Adjuvant therapy with an aromatase inhibitor drug was prescribed for the patient, and she is still alive with no evidence of disease 7 months after surgery.

**Conclusion:**

The awareness of the potential extra-uterine location of ESS should lead to correct diagnosis as this tumor has histopathological features and clinical behavior similar to its uterine counterpart.

## Background

Endometrial stromal sarcoma (ESS) is a rare distinct pathologic entity accounting for only 0.2% of female genital tract tumors [1]. The tumor is commonly found in the uterus, however it can be located elsewhere posing significant diagnostic challenges [1–3]. The extra-uterine ESS (EESS) is supposed to derive from endometriosis, as most reported cases of EESS were associated with foci of endometriosis [2, 4]. Ovaries are common site of EESS, although many organs could be involved, such as peritoneum, vagina, colon, small bowel, stomach, lung [1, 5–9]. In these extra-uterine locations, clinical symptoms are widely variable and misdiagnoses are very common [1]. To claim the diagnosis of a primary EESS, the uterus must be free of tumor as it constitutes the main primary site of ESS [2]. Most reported cases of ovarian ESS were of low grade type, however high grade ovarian ESS have been reported [10].

We report herein a case of a 64-year-old wowan presenting with abdominopelvic and bilateral ovarian tumors diagnosed histologically as low grade ESS arising from ovarian endometriosis.

## Case presentation

In November 2017 a 64-year-old wowan presented to our hospital with abdominopelvic and bilateral ovarian tumors recently discovered on magnetic resonance imaging (MRI). The physical examination was quite normal, the patient did not report metrorrhagia or other gynecologic symptoms. The patient did not report any hormone replacement therapy. Her medical history revealed that she had undergone surgery at an outside hospital for a 18 cm abdominopelvic mass 5 months ago (in June 2017). The patient was also treated for blood hypertension since 2004. At that time, the initial histopathological diagnosis was extra-uterine low grade endometrioid stromal sarcoma (EESS), and the performed endometrial biopsy showed atrophic endometrium with no evidence of tumor. Then, the case has been reviewed by 2 other additional pathologists in different centers, their diagnoses were sex-cord stromal tumor (fibroma) and smooth muscle tumor respectively. Five months later (November 2017), MRI was performed and revealed 2 latero-uterine (ovarian) solido-cystic tumors measuring 60 × 53 mm (left) and 47 × 40 mm (right), along with 2 pelvic masses (located in the recto-vaginal fascia and in the vicinity of the uterine cervix). The uterus was radiologically normal. Then, again the patient underwent subtotal hysterectomy with bilateral salpingo-oophorectomy as well as resection of the 2 pelvic masses and random biopsies of the abdominal wall.

**Fig. 1:**
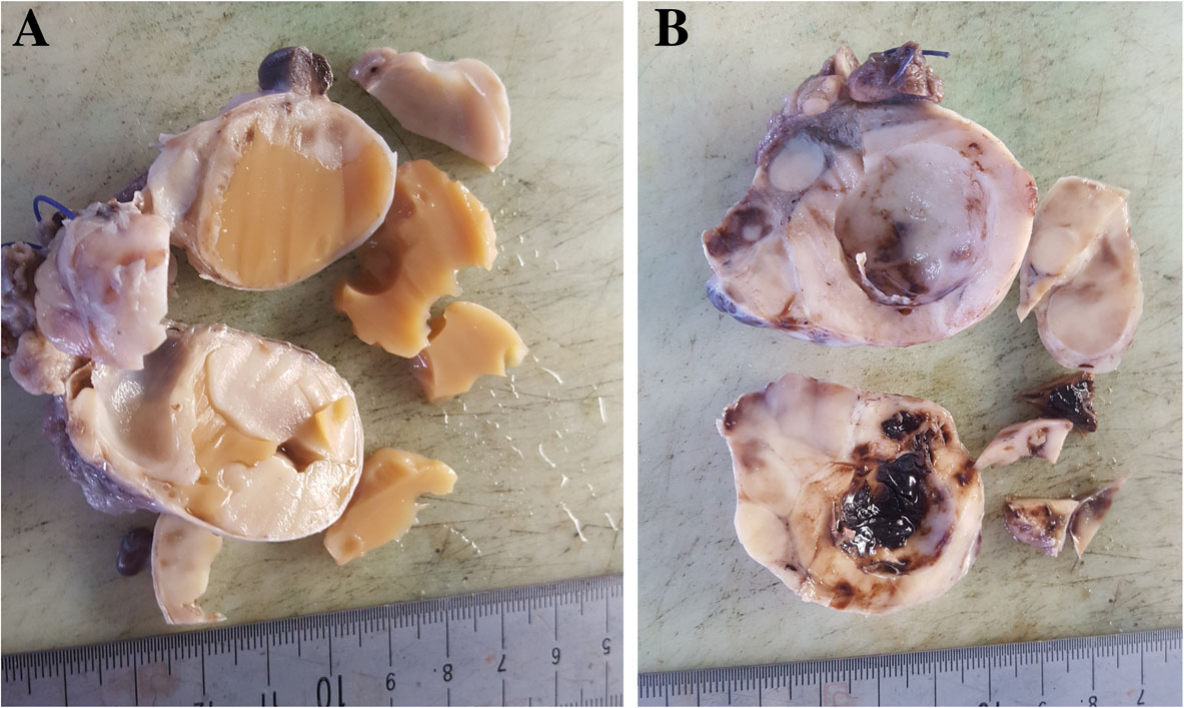
Macroscopic aspects of the ovarian tumors. **a** (right ovary): a well circumscribed solido-cystic tumor, the cut surface showed a vaguely lobulated whitish tumor with cystic areas filled of pasty yellowish material. **b** (left ovary): a whitish lobulated tumor with a cystic areas containing a chocolate-like hemorrhagic material

The macroscopic examination of the resected specimens was as follow:

- Right ovary: a well circumscribed 5 × 4 cm solido-cystic tumor, the cut surface showed a vaguely lobulated whitish tumor with cystic areas filled of pasty yellowish material (Fig. 1a).
- Left ovary: a 6 × 4 cm whitish lobulated tumor with a cystic areas containing a chocolate-like hemorrhagic material (Fig. 1b).
- The 2 pelvic masses: measured 2 × 3 cm and 7 × 8 cm, with solid architecture and pale color.
- Hysterectomy: measured 4 × 5 cm, with no evidence of macroscopic lesion.

**Fig. 2:**
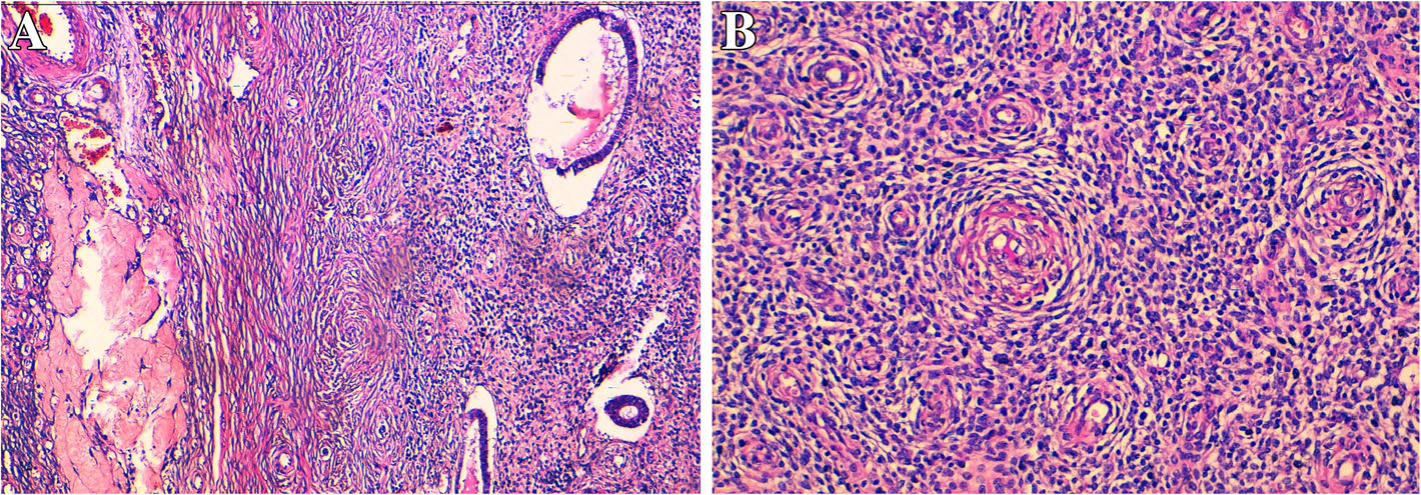
Histologic aspects of the ovarian tumors. **a** (right ovary): the histological image showing ovarian parenchyma infiltrated by a diffuse tumoral proliferation. A focus of endometriosis is shown (Hematoxylin and eosin stain × 100). **b**: the tumor cells are round to spindle with oval hyperchromatic nuclei and moderate cytological atypia. The tumor stroma showed numerous juxtaposed small arterioles with sometimes hyalinazed walls. Tumor cells surrounded these vessels in a striking whorling pattern (Hematoxylin and eosin stain × 200)

**Fig. 3:**
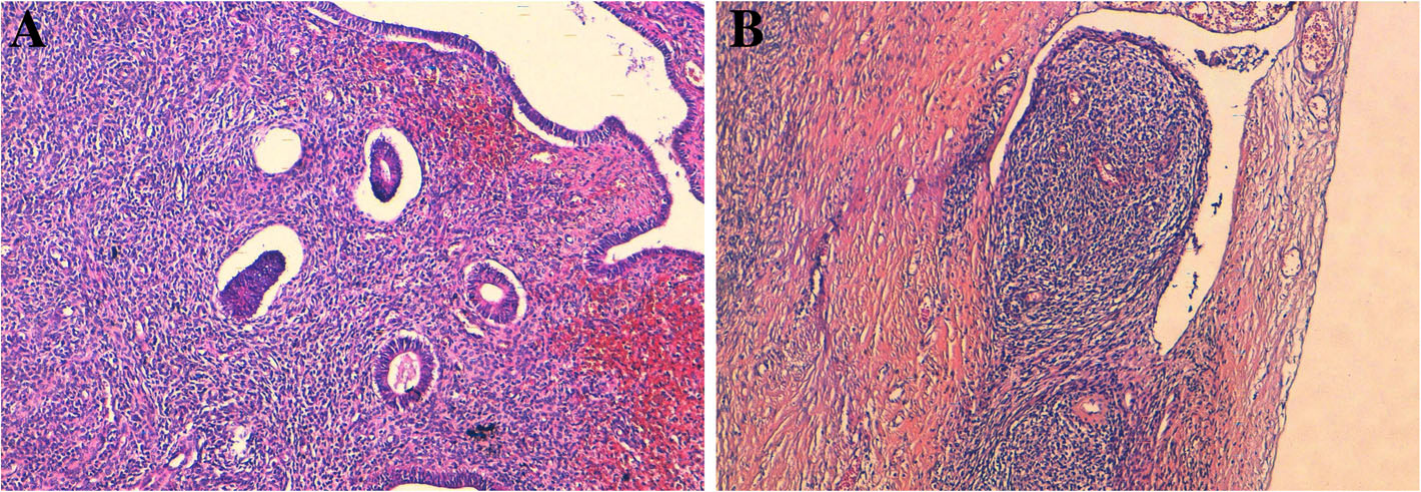
**a** (Endometriosis): a focus of regular dilated endometrioid glands embedded in the tumor (Hematoxylin and eosin stain × 200). **b**: At the peripheral ovarian parenchyma, a tongue-like protrusion in the vessel walls is seen (Hematoxylin and eosin stain × 100)

The histological examination of the right adnexal lesion showed ovarian parenchyma largely occupied by a diffuse tumoral proliferation composed of round to spindle cells with oval hyperchromatic nuclei and moderate cytological atypia, the mitotic figures were scant (3 mitoses/10 high-power fields). The tumor stroma showed numerous juxtaposed small arterioles with sometimes hyalinazed walls. Tumor cells surrounded these vessels in a striking whorling pattern (Fig. 2a and b). In some areas of the tumor (especially cystic areas), foci of regular dilated endometrioid glands were found intimately embedded in the tumor (Fig. 3a). At the periphery of the ovarian parenchyma, a tongue-like protrusion in the vessel walls was observed (Fig. 3b). The histological examination of the other specimens were identical to the right adnexal tumor, however endometrioid glands were not noticed. These histomorphologic characteristics were reminiscent of the proliferative endometrial stroma and the diagnosis of a low grade EESS arising from right ovarian endometriosis was suggested. The examination of the uterus was normal with no evidence of any histological lesion.

**Fig. 4:**
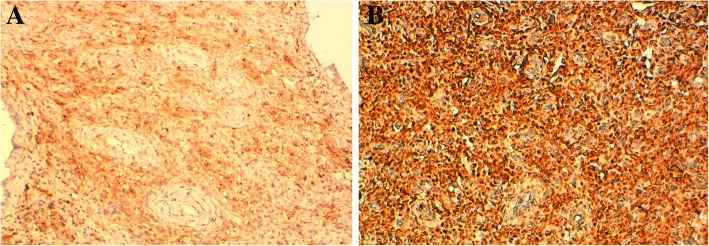
Immunohistochemical features of the tumor (× 200). **a**: tumor cells are positive for CD 10. **b**: tumor cells stain positive for progesterone receptors

**Fig. 5:**
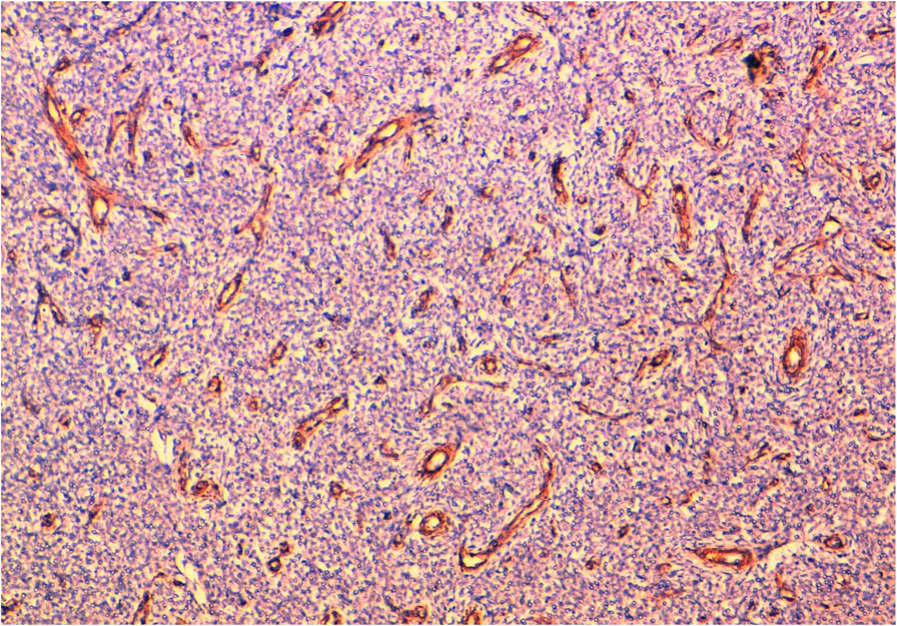
Immunostaining for smooth muscle actin (SMA) is negative in tumor cells, but highlights the tumor stromal rich-vasculature with hyalinazed vessels (× 200)

At immunohistochemistry, tumor cells were positive for CD 10 and for estrogen and progesterone receptors (ER, PR) (Fig. 4a and b), with focal positive staining with desmin. They were negative for smooth muscle actin (SMA) (Fig. 5), inhibin, calretinin and synaptophysin. The diagnosis of disseminated low grade EESS arising from the right ovarian endometriosis was disclosed. Adjuvant therapy with an aromatase inhibitor drug (letrozole) was performed, and the patient is still alive with no evidence of disease 7 months after surgery.

## Discussion

Extra-uterine endometrioid stromal sarcoma (EESS) is an extremely uncommon entity as the current literature offers only some case reports and short series [1, 2, 4, 10]. In 2013, Masand et al. reported the largest series of EESS with 63 cases, ovarian involvement was found in 25 patients [1]. Ovaries are the most common site of EESS, the patients’ age ranged from 34 to 76 years with a median age around 50 years [2, 10]. Foci of endometriosis are frequently found embedded within EESS and these tumors are thought to arise from endometriosis [1, 4]. The clinical presentation of EESS is not specific, it is often related to the tumor location and size. Reported cases of patients with ovarian ESS presented with wide clinical symptoms, from asymptomatic to abdominal distension [2, 10]. Mostly, ovarian ESS was diagnosed in advanced stages with tumor extension beyond the ovaries, especially in the pelvis or abdomen [2, 4, 10]. Our current case typically illustrates this clinical feature as previously reported in the literature; she presented initially with a huge abdominopelvic mass, then 5 months later with radiologically discovered ovarian and pelvic tumors. However, because of the initial absence of ovarian tumors, one could speculate that our patient had perhaps abdominopelvic foci of endometriosis that had given rise to the abdominopelvic tumors, along with bilateral ovarian tumors. The presence of endometriosis foci in the right ovarian tumor of our patient favors at least its primary nature at this site, and we could not speculate about the true nature of the initial abdominopelvic tumor as it had been diagnosed elsewhere outside our hospital. Also, all these abdominopelvic tumors could be metastases from the right ovarian ESS clinically and radiologically missed out at the initial evaluation of our patient. However, the fact that we have no idea about the initial tumor of our patient does not affect the accuracy of our current histological diagnosis. In the literature cases of disseminated EESS with misleading clinical presentation have been reported, Mourra et al. have reported a case of a rectosigmoid ESS presenting with epigastric pain due to portal vein thrombosis [11].

The definitive diagnosis of ESS relies on pathology as imaging techniques do not provide specific signs. In fact the histologic diagnosis of low grade ESS is often straightforward when in uterine location, challenges arise when the tumors are found in inhabitual extra-uterine locations [1–3, 12]. Typically, ESS presents as a neoplasm that resembles proliferative phase endometrial stroma, with diffuse architecture and monomorphic cells with oval to spindle nuclei; mitotic count is variable and this criterion is no longer considered by the current World Health Organisation (WHO) classification of tumors of female reproductive organs [3, 12]. The tumor stroma has a rich vascular network of small vessels sometimes with hyalinized walls, reminiscent of endometrial spirale arterioles, and the tumor cells are frequently arranged in a whorling pattern around these vessels [1, 3, 12]. Sometimes inhabitual features of ESS could be found: smooth muscle differentiation, myxoid background, fibroblastic appearance, calcifications, epithelioid differentiation, sex cord-like differentiation, clear cells differenciation, ...etc. [1–3]. The tumor borders are usually irregular with vascular invasion and tongue-like projections into vessels wall. At immunohistochemistry, typically ESS stains positive for CD10, vimentin, WT-1, ER, PR, and negative for SMA, desmin, CD34, CD31, inhibin, calretinin [3, 12]. However, areas of sex-cord differenciation stain positive for inhibin and calretinin, also smooth muscle differenciation areas are positive for smooth muscle immunomarkers (SMA, desmin) [12]. The most common genetic abnormality in low-grade ESS is t(7,17)(p15;q21) resulting in the fusion of JAZF1 and SUZ12 (JJAZ1) genes at 7p15 and 17q21 respectively [3, 13]. However Amador-Ortiz et al. have recently shown that this genetic abnormality is rarely found in their 6 reported cases of EESS (1 case out of 6) [14].

The inhabitual locations of ESS (extra-uterine sites) make the diagnosis very challenging for both clinicians and pathologists. In their series of EESS, Masand et al. reported that initial misdiagnoses were: ovarian stromal neoplasm, leiomyosarcoma, gastrointestinal stromal tumor (GIST), adult granulosa cell tumor, juvenile granulosa cell tumor, liposarcoma, small round blue cell tumor, adenosarcoma, cellular fibroma, malignant peripheral nerve sheath tumor, atypical stromal endometriosis, and poorly differentiated synovial sarcoma [1]. A part from the extra-uterine locations, these erroneous diagnoses could be in part due to many changes that occurred in histologic classification of ESS during recent years. Our case has been misdiagnosed initially as sex stromal tumor and as smooth muscle tumor. In fact, cases of ESS with unusual morphologic features (sex-cord differention, smooth muscle differentiation,...) pose differential diagnoses with sex-cord stromal tumors or smooth muscle tumors. However, these unusual features are often focal in ESS, and the characteristic diffuse architecture with striking vascular-rich stroma with whorling pattern should lead to correct diagnosis. Ovarian ESS with spindle cells could be mistaken for metastases from GIST or other sarcoma [2]. A minimal immunohistochemical panels can easily rule out these differential diagnoses: inhibin, calretinin positive in sex-cord stromal tumors and negative in ESS, muscle markers (SMA, desmin, caldesmone) positive in smooth muscle tumors, CD117 and DOG-1 positive in GIST and negative in ESS. Another differential diagnosis of EESS is metastasis from a primary uterine ESS. To claim the diagnosis of EESS, the status of the uterus should be determined by imaging techniques or by a thorough macroscopic sampling when the uterus is resected [1, 2]. Our patient had no clinical, radiological or macroscopic evidence of any uterine lesions and histomorphologic features were characteristic of low grade ESS. The immunohistochemical phenotype was also compatible with ESS (positivity of ER, PR, CD10).

The low grade extra-uterine endometrioid stromal sarcoma (EESS) is considered as an indolent neoplasm with a propensity for late recurrences despite the fact that patients frequently presented with advanced tumor stages [1, 2, 10]. The therapeutic management is not well defined due to the rarity of EESS, surgical treatment is the ideal option however adjuvant therapy (hormonal therapy, chemotherapy or radiation therapy) should be considered in patients with advanced tumor stages [1, 10].

We have found only 89 reported cases of primary ovarian ESS in the English literature [1, 2, 4, 10, 14–29]. Table 1 summerises some features of these reported cases of ovarian ESS. The histopathologic terminology and diagnostic criteria for ovarian ESS have greatly changed across years, making very approximative any attempt to conduct a precise retrospective literature review. Endometrioid stromal sarcoma has been designated previously as stromal endometriosis [15, 16], endometrial stromatosis [18] or endolymphatic stromal myosis [20]. The mean age of our 90 cases (previous cases and our current case) of primary ovarian ESS is 46.62 years (range of 34–65 years). Most patients presented with metastases (67 patients, 74.44%), the tumor was bilateral in 29 cases (32.22%), left-sided in 22 cases (24.44%), right-sided in 22 patients (24.44%) while the tumor location was not available in 17 cases (18.88%). The average tumor size was 9.42 cm (range of 4–15 cm), endometriosis was found in 51 patients (56.66%). Fourty one patients (45.55%) were treated by surgery alone, 36 cases (40%) were treated by surgery associated with chemotherapy or hormonal therapy; radiation therapy was associated to surgery in 4 cases (4.44%). The follow-up duration ranged from 5 weeks to 24 years. Fifty one patients (56.66%) were alive with no evidence of disease, 11 (12.22%) were alive with disease and 12 cases (13.33%) were died of disease whereas follow-up data were not availabe in 16 cases (17.77%).

**Table 1:** Reported cases of ovarian endometrioid stromal sarcoma

References	No of cases	Age/Yr	Laterality	Size	Endom.	Metastasis	Treatment	Follow-up
Koller and Rygh [15]	1	56	Left	NA	+	+	S + R	NED (15 Mo)
Benjamin and Campbell [16]	1	37	Bilateral	6 cm	+	+	S	NED (5 weeks)
Palladino and Trousdell [17]	1	42	Bilateral	NA	+	–	S	DOD (16.5 yr)
Gruskin et al. [18]	1	47	Left	15 cm	+	–	S	NED (1 yr)
Azoury and Woodruff [19]	2	41*	Left (1 case) Right (1 case)	NA	+ (2 cases)	+ (1 case)	S + R	DOD (2 yr) NED (24 yr)
Silverberg and Fernandez [20]	3	48*	Left (2 case) Right (1 case)	9.33 cm*	+ (2 cases)	+ (3 cases)	S (2 cases) S + M (1 case)	NED (3 cases; 3.16 yr.*)
Baiocchi et al. [21]	1	50	Left	12 cm	+	+	S + M	NED (10 Mo)
Fukunaga et al. [22]	1	40	Bilateral	15 cm	+	+	S + M	NED (16 Mo)
Mitchard et al. [23]	1	35	Right	4 cm	+	+	S	NA
Geas et al. [24]	1	45	Bilateral	15 cm	+	+	S + M	NED (36 Mo)
Kim et al. [25]	1	50	Bilateral	6 cm	+	+	S + M + R	AWD (3 Mo)
Lan et al. [26]	2	45.5*	Left (1 case) Right (1 case)	NA	+ (2 cases)	+	S + M (2 cases)	NED (2 cases; 8.5 yr.*)
Amador-Ortiz et al. [14]	3	42^*^	Right (1 case) Left (1 case) Bilateral (1 case)	NA	+ (2 cases)	NA	NA	NA
Masand et al. [1]	25	50.56*	1 ov (15 cases Bilateral (10 cases)	NA	+ (9 cases)	+ (22 cases)	S (7 cases) S + M (16 cases) S + R (2 cases)	NED (17 cases; 78,17 Mo*) AWD (2 cases; 33 Mo*) DOD (1 case; 228 Mo*) NA (5 cases)
Oliva et al. [2]	27	56*	Right (9 cases) Left (8 cases) Bilateral (8 cases) NA (2 cases)	9.5 cm*	+ (16 cases)	+ (20 cases)	S (25 cases) S + M (1 case) S + M + R (1 case)	NED (10 cases; 10,3 yr.*) AWD (5 cases; 13 yr.*) DOD (6 cases; 6,8 yr.*) NA (6 cases)
Back et al. [4]	1	40	Bilateral	6 cm	+	+	S + M	NED (14 Mo)
Kikuchi et al. [27]	1	65	Right	12 cm	–	+	S + M	DOD (2 Yr)
Xie et al. [10]	14	49.1^*^	Right (7 cases) Left (4 cases) Bilateral (3 cases)	9.5 cm*	+ (6 cases)	+ (8 cases)	S (2 cases) S + M (10 cases) S + M + R (2 cases)	NED (9 cases) AWD (3 cases) (65 Mo*) DOD (2 cases)
Ilanthodi et al. [28]	1	34	Left	11 cm	+	+	S + M + R	NA
Wang et al. [29]	1	42	Left	5 cm	–	+	S	NED (10 Mo)
Our case	1	64	Bilateral	6 cm	+	+	S + M	NED (7 Mo)

*= average; + = present; − = absent; *ov* = ovary, *yr.* = year, *Mo* = month, *Endom.* = endometriosis, *S* = surgical treatment, *M* = medical treatment (chemotherapy and/or hormonal therapy), *R* = radiation therapy, *NA* = not available, *NED* = no evidence of disease, *AWD* = alive with disease, *DOD* = died of disease

## Conclusions

Extra-uterine low grade endometrioid stromal sarcoma (EESS) is an extremely rare tumor with misleading clinical presentation and diagnostic challenge. The awareness of the potential extra-uterine location of this low grade tumor should guide clinicians and pathologists to the correct diagnosis as EESS has histopathological features and clinical behavior similar to its uterine counterpart.

## Abbreviations

EESS: Extra-uterine endometrioid stromal sarcoma
ER: Estrogen receptors
ESS: Endometrial stromal sarcoma
GIST: Gastrointestinal stromal tumor
MRI: Magnetic resonance imaging
PR: Progesterone receptors
SMA: Smooth muscle actin
WHO: World health organisation
